# On the relationship between conspiracy theory beliefs, misinformation, and vaccine hesitancy

**DOI:** 10.1371/journal.pone.0276082

**Published:** 2022-10-26

**Authors:** Adam M. Enders, Joseph Uscinski, Casey Klofstad, Justin Stoler

**Affiliations:** 1 Department of Political Science, University of Louisville, Louisville, Kentucky, United States of America; 2 Department of Political Science, University of Miami, Coral Gables, Florida, United States of America; 3 Department of Geography and Sustainable Development, University of Miami, Coral Gables, Florida, United States of America; University of Padova, ITALY

## Abstract

At the time of writing, nearly one hundred published studies demonstrate that beliefs in COVID-19 conspiracy theories and misinformation are negatively associated with COVID-19 preventive behaviors. These correlational findings are often interpreted as evidence that beliefs in conspiracy theories and misinformation are exogenous factors that shape human behavior, such as forgoing vaccination. This interpretation has motivated researchers to develop methods for “prebunking,” “debunking,” or otherwise limiting the spread of conspiracy theories and misinformation online. However, the robust literatures on conspiracy theory beliefs, health behaviors, and media effects lead us to question whether beliefs in conspiracy theories and misinformation should be treated as exogenous to vaccine hesitancy and refusal. Employing U.S. survey data (n = 2,065) from July 2021, we show that beliefs in COVID-19 conspiracy theories and misinformation are not only related to COVID-19 vaccine hesitancy and refusal, but also strongly associated with the same psychological, social, and political motivations theorized to drive COVID-19 vaccine hesitancy and refusal. These findings suggest that beliefs in conspiracy theories and misinformation might not always be an exogenous cause, but rather a manifestation of the same factors that lead to vaccine hesitancy and refusal. We conclude by encouraging researchers to carefully consider modeling choices and imploring practitioners to refocus on the worldviews, personality traits, and political orientations that underlie both health-related behaviors and beliefs in conspiracy theories and misinformation.

## Introduction

Since the beginning of the COVID-19 pandemic, conspiracy theories and misinformation (CTM) have been a prime concern of researchers across disciplines [1–3], and for good reason: a wealth of research consistently demonstrates a strong negative relationship between beliefs in pandemic-related CTM and disease-preventive behaviors, including vaccine refusal [for review, see 4]. Thus, many scholars have come to believe that the spread of CTM represents a “crisis situation” [5] or “infodemic” as dangerous as the pandemic itself [6]. One characteristic of this research is the assumption of a specific causal link: that beliefs in CTM *cause* vaccine hesitancy and refusal.

This assumption has become influential in discussions of COVID-19 CTM. It has motivated the development of methods for pre-bunking [7] and debunking [8] beliefs in CTM, and has led researchers to call for the “downgrading, blocking, and counteracting” of online CTM [9]. Likewise, journalists often blame CTM for deleterious beliefs and behaviors [10] and U.S. lawmakers have proposed legislation to limit CTM online [11]. U.S. President Joe Biden, for example, claimed that online CTM was responsible for “killing people” [12].

While beliefs in CTM are undisputedly correlated with vaccine hesitancy and refusal, we question the frequent interpretation of such correlations as evidence of the causal, exogenous impact of CTM. As Scheufele et al. [13] recently argued, for example, the prevailing assumption that CTM “significantly distorts attitudes and behaviors of a citizenry that would otherwise hold issue and policy stances that are consistent with the best available scientific evidence” has “limited foundations in the social scientific literature.” Instead, we argue, and demonstrate empirically, that it may also be the case that, for many Americans, CTM are endogenous to vaccine hesitancy and refusal––i.e., they are less so unique causal explanations for vaccine hesitancy than they are beliefs reinforced by vaccine hesitancy or caused by underlying factors shared with vaccine hesitancy. This is primarily because the psychological, social, and political motivations––as well as situational environmental factors––that promote vaccine-related attitudes and behaviors have also been found to promote beliefs in CTM. For example, a lack of trust in the scientific community may cause individuals to eschew the COVID-19 vaccine and concurrently to adopt beliefs in CTM.

Our argument is built on the foundational literatures addressing media effects, beliefs in CTM, and health-related beliefs and behaviors, which suggest that 1) beliefs in CTM are likely to be endogenous to vaccine attitudes and behaviors, and that 2) exposure to CTM in the information environment does not necessarily promote beliefs in CTM. To illustrate the plausibility of our argument empirically, we employ nationally-representative U.S. survey data from July 2021 (n = 2,065) measuring beliefs in COVID-19 CTM and vaccine hesitancy and status. A factor analysis shows that beliefs in COVID-19 CTM and vaccine hesitancy can be conceived of as sharing a single dimension of opinion and that these phenomena are sufficiently correlated that distinguishing between them is difficult. Next, we explore the role of COVID-19 CTM in the theoretical and empirical models typically used to explain vaccine hesitancy and refusal. We demonstrate that the characteristics that predict belief in COVID-19 CTM also predict vaccine status and hesitancy, thereby establishing the plausibility of the argument that beliefs in CTM are, at least partially, endogenous to vaccine hesitancy.

Our argument attempts to reconcile two growing bodies of literature: one expressing alarm about the role of CTM in driving deleterious attitudes and behaviors [5,9,14,15] and another expressing doubt about the influence of CTM, which suggests that its influence on attitudes and behaviors is limited and conditional [13,16–19]. Simply put, we argue that the characteristics that foster exposure and acceptance of CTM need to be properly accounted for. When failing to account for these factors, scholars may inappropriately make causal claims that potentially misattribute the foundations of vaccine hesitancy and, consequently, misdirect efforts at increasing vaccination rates.

## The connection between COVID-19 CTM and vaccine hesitancy and refusal

Since the onset of the COVID-19 pandemic, scholars have focused on the circulation of CTM about the virus’s origins, effects, and treatments, as well as the potential impact of CTM on pandemic-related behaviors [20]. The vast majority of these studies––across different disciplines and focusing on various countries––demonstrate that beliefs in COVID-19 CTM have a strong negative association with pro-social and disease-preventative health behaviors [see review in 4].

The most protective COVID-19 public health measure individuals can partake in is vaccination, yet vaccination rates in many parts of the world have stagnated. Numerous studies demonstrate a negative association between beliefs in COVID-19 CTM and vaccine intentions and status [e.g., 21–23], providing a potential clue about why.

Despite the correlational nature of these studies, negative relationships between beliefs in COVID-19 CTM and COVID-19 vaccine attitudes and behaviors are typically interpreted through a particular causal lens: that vaccine hesitancy is, at least partially, *caused* by (online) exposure to and belief in CTM [15,24,25]. For example, based on cross-sectional, correlational evidence, Allington et al. [15] argue (*emphasis added*) that “conspiracy beliefs *act to* inhibit health-protective behaviours and that social media *act as* a vector for such beliefs.” Dow et al. argue that “*social media radicalizes beliefs*, increasing contagion (rapid spread) and stickiness (resistance to change) of conspiracy theories. As conspiracy theories are reinforced in online communities, social norms develop, *translating conspiracy beliefs into real-world action*.” Dow et al. further argue that “COVID-19 *conspiracy beliefs translate directly to various health-protective behaviors*,” and that “*conspiracy beliefs reduce healthy behaviors*” [14]. Goreis and Kothgassner contend that the “*endorsement of such theories reduces compliance with mandated measures*” [24]. The scholarly literature also implies such a relationship when certain causal processes are assumed. For example, Romer and Jamieson conclude that “it will be critical to confront both conspiracy theories and vaccination misinformation to prevent further spread of the virus in the US” [20].

**Fig 1:**
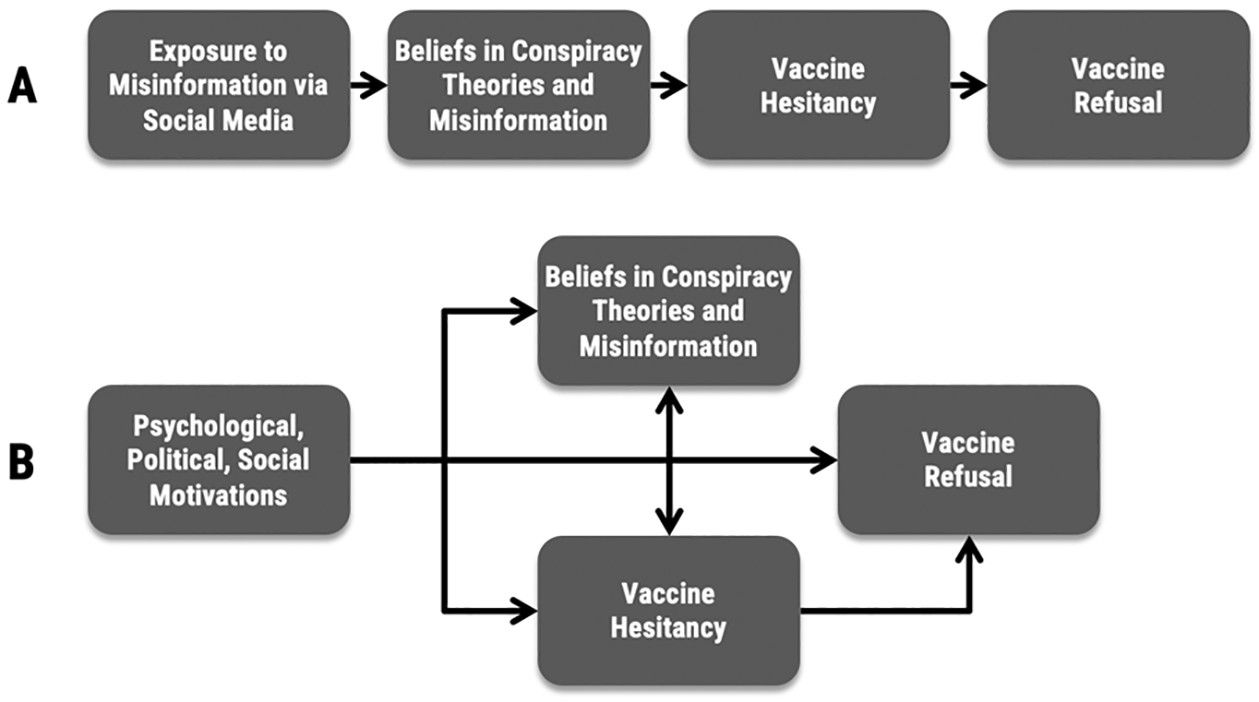
Two conceptual models of relationship between beliefs in COVID-19 conspiracy theories and misinformation and vaccine hesitancy and refusal.

The conceptual model in Panel A of Fig 1 details the relationship between beliefs in COVID-19 CTM and vaccine hesitancy and refusal assumed by many scholars, journalists, and policymakers [e.g., 26]: exposure to COVID-19 CTM vis-à-vis media use––especially social media use––leads to beliefs in COVID-19 CTM, which subsequently result in vaccine hesitancy, and finally vaccine refusal. Under this and similar models, the obvious prescription for boosting vaccination rates involves limiting CTM in the information environment [9], or perhaps inoculating people against such ideas in advance of exposure [7]. Thus, researchers have recommended “aggressive” actions [9] because “unregulated social media may present a health risk” [15]. However, two robust bodies of literature provide reasons for skepticism of the presumed causal pathway depicted in Panel A of Fig 1.

First, a wealth of research on the causal antecedents of beliefs in CTM conceives of such beliefs as the product of deep-seated psychological, social, and political motivations [27], with exposure sometimes playing a more limited and conditional role [28]. Thus, beliefs in CTM could be treated as the downstream products of more foundational characteristics, rather than as explanatory factors in and of themselves. This theoretical perspective follows widely-accepted and well-evidenced models of both health and political belief formation: people’s worldviews, ideologies, values, group attachments, personalities, and even occasionally biological and genetic factors––the relatively stable features of our identities and belief systems––are the primary ingredients of beliefs regarding politics, health and medicine, and various forms of CTM [29–31]. In other words, beliefs in COVID-19 CTM and pandemic-related behaviors share many underlying (presumably causal) ingredients [32].

For example, conspiracy mentality [33,34], scientific literacy and trust in science [35,36], political ideology and partisan attachments [20,37], social media use [9], personality traits [38,39], and emotional conditions, such as stress and anxiety [40,41], have been found to predict *both* beliefs in COVID-19 CTM and pandemic-related behavioral intentions. Moreover, whereas Panel A of Fig 1 suggests a recent cause for beliefs in COVID-19 CTM and vaccine refusal (i.e., exposure to COVID-19 CTM), vaccine CTM and vaccine hesitancy, more generally, are hardly new problems. Previous vaccines have faced longstanding opposition [42], and the factors associated with opposition to vaccines, in general, are also associated with opposition to COVID-19 vaccines, specifically [43–45]. Thus, refusing to vaccinate for COVID-19 may be related to motivational factors that preexist not only exposure to COVID-19 CTM but also the COVID-19 pandemic itself.

These points come into focus when considering the implied influence of information exposure detailed in Panel A of Fig 1. The causal influence of exposure to COVID-19 CTM by traditional and social media, or from elsewhere, suggests a “hypodermic needle” effect [46], whereby (incidental) exposure directly promotes belief. However, a century-long literature demonstrates that media effects are more limited, conditional on individual and environmental factors, and, most importantly, partially endogenous to beliefs themselves [47–53].

Second, selective exposure and avoidance––the tendency for individuals to seek out information that comports with previously held beliefs or ignore that which is incongruent with said beliefs [54,55]––are better-evidenced models of media effects than the hypodermic model. Selective exposure also guides the search for/avoidance of and acceptance/rejection of CTM, specifically: those drawn to conspiracy theories tend to seek them out, and individuals not attracted to conspiratorial explanations avoid them [56]. The same can be said of vaccine skeptical content: very few people not exhibiting vaccine hesitant attitudes choose to consume vaccine skeptical content [57]. Even for individuals who are incidentally exposed to CTM, previously held dispositions and beliefs will temper persuasion effects through processes such as motivated reasoning [58]. Thus, we possess additional reasons to expect that exposure to CTM––regardless of medium––might serve more as an expression of, reinforcement for, or rationalization for previously held beliefs, values, and predispositions, rather than an impetus for individual change.

Considering these bodies of past work, we propose an alternative theoretical model of vaccine hesitancy and refusal in Panel B of Fig 1 in which pre-existing social, political, and psychological motivations are key to understanding vaccine hesitancy and refusal. This model, while containing many of the same elements as Panel A, better incorporates past theories and empirical findings regarding the causes of beliefs about CTM and vaccines [59]. In this model, both beliefs in COVID-19 CTM and vaccine hesitancy are promoted by social, political, and psychological motivations; those motivations in Panel B largely replace the assumed foundational role of exposure to COVID-19 CTM vis-à-vis exposure in Panel A. For example, those who distrust doctors and scientists are more likely to concurrently hold vaccine hesitant attitudes *and* believe in COVID-19 CTM. This not only better incorporates the literature on the foundations of beliefs in CTM and vaccine hesitant attitudes, but more accurately accounts for well-evidenced theories of media effects.

The model in Panel B also allows for a reinforcing relationship between beliefs in COVID-19 CTM and vaccine hesitancy. Because of the tendency for individuals to reinforce their beliefs and identities, vaccine hesitancy might encourage or further strengthen beliefs in CTM, just as beliefs in CTM may encourage or further strengthen vaccine hesitancy. This reciprocal relationship allows for the possibility of beliefs in COVID-19 CTM to be adopted *after* an individual is vaccine hesitant, a scenario in which CTM serve as a rationalization for, rather than a motivating force behind, attitudes and behaviors. This process comports with scholarship showing that people will often adopt beliefs in CTM to justify pre-existing views in the face of disconfirming evidence or use CTM to rationalize personal or political losses [60]. Further, Panel B is consistent with individuals who base their vaccine hesitancy on a stylized interpretation of authoritative facts (e.g., the COVID-19 vaccine is ‘not yet fully approved’) [61] and with pro-vaccine individuals who share beliefs in various CTM [62]. Exposure to, and beliefs in, COVID-19 CTM in Panel B are, therefore, neither necessary nor sufficient for the adoption of vaccine hesitant attitudes or vaccine refusal.

Before exploring the plausibility that a different process (Panel B) underlies vaccine hesitancy/refusal than is commonly assumed (Panel A), we wish to make several points clear. Most importantly, we are not arguing that the typical model (Panel A) is flatly incorrect and that the proposed alternative (Panel B) is correct. Indeed, it strikes us as highly likely that beliefs in CTM are neither strictly exogenous nor completely endogenous to vaccine hesitancy––the world is complicated and filled with reciprocal effects promoted by changes in situational factors and other contextual considerations. Rather, we argue only that the alternative model (Panel B) is *plausible*, *given the observational nature of the vast majority of analyses (including our own) and past findings regarding media effects and the predictors of beliefs in CTM and vaccine hesitancy pre-pandemic*. Our intention is to contribute a different perspective to the conversation on the relationship between CTM and vaccine hesitancy and refusal, not to dismiss past work that reliably finds correlational associations between these constructs.

### Expectations

If the alternative model we propose is *plausible*, we expect to observe several patterns in our analyses. First, an analysis of the dimensionality of beliefs in CTM and vaccine hesitancy (e.g., using factor analysis) will reveal a unidimensional structure. This would suggest that beliefs in CTM and vaccine hesitancy are more similar than different, perhaps because of the social, political, and psychological antecedents that they share (though this is not the only possibility, which we discuss below). Second, an analysis of the predictors of beliefs in CTM, on the one hand, and vaccine hesitancy and refusal, on the other, should reveal that both sets of dependent variables share many of the same correlates. This, too, would provide evidence for the *plausibility* that beliefs in CTM are not strictly exogenous to vaccine hesitancy and refusal, as has often been taken for granted in the literature.

## Materials and methods

Our central argument is that beliefs in COVID-19 CTM should not, based on current evidence, be thought of as strictly exogenous to vaccine hesitancy and behavior. It is just as plausible that beliefs in CTM are endogenous to vaccine hesitancy. Therefore, our empirical examination is designed to demonstrate the *plausibility* of Panel B of Fig 1. We utilize a unique national survey containing questions about COVID-related CTM, as well as questions designed to measure a wealth of psychological, social, and political traits and orientations previously found to promote beliefs in CTM [27]. These include science literacy, trust in scientists and health professionals, Machiavellianism, narcissism, psychopathy, perceived victimhood, stress, conflictual behavior, conspiracy thinking, partisanship, ideology, support for Donald Trump, and social media usage, in addition to age, educational attainment, religiosity, perceived socioeconomic status, race and ethnicity, and gender. While this dataset was initially collected in the context of a project on deciphering the various correlates of vaccine hesitancy, it is also capable of aiding in the exploration of the plausibility of the alternative model we pose because it contains many of the focal predictors of both beliefs in CTM and vaccine hesitancy. Details regarding question wording, summary statistics, and scale reliability appear in the Supplementary Information (SI); all variables are captured using previously developed and validated measures.

We partnered with Qualtrics to interview 2,065 U.S. adults between July 17–August 5, 2021. The sample was designed to match the 2019 U.S. Census American Community Survey records on sex, age, race, and education; details about the sociodemographic composition of the sample appear in the SI. This research was approved by the University of Miami Human Subject Research Office on July 14, 2021 (Protocol #20210618). In order to ensure quality, respondents were required to pass four attention checks; these included a mixture of standalone questions and questions embedded in grids, per best practices [63]. Participants who failed to pass all four attention checks were excluded from the data set. Qualtrics also took steps to eliminate “speeders”––respondents who quickly completed the survey without reading the questions. A soft-launch of the survey (n = 127) yielded a median time to complete of 11.6 minutes. Participants completing the questionnaire in less than half the median time were excluded from the final n = 2,056 dataset delivered to us by Qualtrics.

The specific beliefs that comprise our CTM measure appear in Table 1, along with the percentage of respondents who either “agree” or “strongly agree” with each. We observe significant variability in the popularity of these ideas. Both sets of questions were averaged into additive scales of beliefs in conspiracy theories (α = 0.90, Range = 1–5, *M* = 2.20, *SD* = 1.04) and beliefs in misinformation (α = 0.93, Range = 1–5, *M* = 2.16, *SD* = 1.06).

**Table 1:** Proportion of Americans who believe in COVID-19 conspiracy theories and misinformation.

Conspiracy/Misinformation Belief Question (*Label*)	% Agree
**COVID-19 Vaccine Misinformation Beliefs**	
1. The COVID-19 vaccine can give you COVID-19. (*Item 1*)	18
2. The COVID-19 vaccine is a scam by the pharmaceutical companies to make money. (*Item 2*)	15
3. The COVID-19 vaccine will alter your DNA. (*Item 3*)	12
4. The COVID-19 vaccine causes infertility. (*Item 4*)	11
5. People receiving the COVID-19 vaccine will "shed" dangerous chemicals from that vaccine. (*Item 5*)	11
**COVID-19 Conspiracy Beliefs**	
1. The number of deaths related to the coronavirus has been exaggerated. (*Item 1*)	29
2. Coronavirus was purposely created and released as part of a conspiracy. (*Item 2*)	25
3. The coronavirus is being used to force a dangerous and unnecessary vaccine on Americans. (*Item 3*)	20
4. The coronavirus is being used to install tracking devices inside our bodies. (*Item 4*)	12
5. Bill Gates is behind the coronavirus pandemic. (*Item 5*)	11
6. 5G cell phone technology is responsible for the spread of the coronavirus. (*Item 6*)	9

Note: Percentages calculating using “agree” and “strongly agree” responses.

We utilize two dependent variables. The first is self-reported vaccination status. We asked respondents: “Have you personally received at least one dose of a COVID-19 vaccine?” Sixty-eight percent reported having received the vaccine, 32 percent reported not having received it. We also asked those who reported not having received the vaccine: “Which of the following best describes how you feel about the COVID-19 vaccine?” Response options included: “I plan to get the vaccine as soon as possible” (12%), “I am open to it, but will keep waiting and see what happens” (25%), “I will only get the vaccine if required by my job or places I need to go” (12%), and “I definitely will not get the vaccine” (51%). For this dependent variable, we coded as 1 those who received the vaccine and those who reported a plan to get the vaccine as soon as possible (72%); those who had not received the vaccine and were either unwilling or had no immediate intention of being vaccinated (28%) are coded 0. We also estimated the models below using only the initial question about vaccination status where only those who have not been vaccinated and are completely unwilling to be vaccinated are coded as 0; results, which are available in the SI, are substantively identical to those provided below.

Our second dependent variable is an additive scale of attitudes about vaccines [64], coded such that greater values reflect stronger vaccine hesitancy (α = 0.91, Range = 1–5, *M* = 2.34, *SD* = 0.83). For example, respondents were asked to what extent they agreed with the following: “Getting vaccines is a good way to protect me from disease” and “I do not need vaccines for diseases that are not common anymore.” The correlation between this measure of vaccine hesitancy and vaccination status is -0.62 (*p*<0.001).

### A note on causality

The data we employ is cross-sectional and, therefore, unsuited for demonstrating causality. We reiterate that our objective is to illustrate the *plausibility* of our argument that beliefs in CTM are (at least partially) endogenous to vaccine hesitancy and refusal, rather than strictly exogenous. While, at first glance, mere plausibility may seem underwhelming, this is precisely the same standard that most published studies on CTM and vaccine hesitancy are capable of meeting, even though a particular causal interpretation is frequently––and, perhaps, inappropriately––employed or assumed. In other words, our study is not unique in utilizing cross-sectional data––it is unique only by way of our explicit attention to the fact that our data are incapable of establishing unidirectional causal relationships.

This is not to say that other, “better” designs are impossible (e.g., experiments, panel data). We merely highlight that most studies, like our own, are only capable of revealing the plausibility of an argument. Our intent is to demonstrate the plausibility of an argument that has been, to the detriment of the relevant literatures and practical efforts to combat vaccine hesitancy, largely overlooked.

## Results

### Assessing the dimensionality of beliefs

We begin our analysis by examining the results of an exploratory factor analysis of the items composing the conspiracy theory, misinformation, and vaccine hesitancy scales described above. If beliefs in CTM and vaccine hesitancy are the related to shared social, psychological, and political antecedents, it stands to reason that they may occupy a shared dimension of opinion, rather than three distinct dimensions each representing conspiracy theories, misinformation, and vaccine hesitancy. Before estimating the factor model, we conducted Horn’s parallel analysis for guidance on the appropriate number of factors to retain; this analysis suggested that two factors are appropriate. We also examined a scree plot (see SI), which prompted the same conclusion (the “elbow” appearing at the third factor suggests a two-factor solution).

We estimated the two-factor solution using iterated principal axis factoring; (unrotated) factor loadings and other details appear in Table 2. We bolded the greatest loading in absolute value associated with each item; note that the exact wording for each item appears in 1. In each case, the greatest loading is associated with the first factor, usually by a considerable discrepancy. The eigenvalue associated with the first factor is also approximately six times greater than that associated with the second factor. These results suggest that vaccine hesitancy can be conceived as occupying the same dimension of opinion as beliefs in COVID-19 CTM.

**Table 2:** Exploratory factor analysis results.

Item	Factor 1	Factor 2	Uniqueness
**Vaccine Hesitancy**			
Item 1	**-0.737**	0.441	0.263
Item 2	**-0.731**	0.359	0.337
Item 3	**-0.759**	0.398	0.266
Item 4	**-0.680**	0.421	0.361
Item 5	**-0.565**	-0.101	0.671
Item 6	**-0.682**	0.366	0.401
Item 7	**-0.741**	0.413	0.281
Item 8	**-0.612**	0.426	0.445
Item 9	**-0.537**	-0.076	0.706
Item 10	**-0.620**	-0.032	0.614
**Conspiracy Beliefs**			
Item 1	**0.664**	0.139	0.540
Item 2	**0.695**	0.198	0.478
Item 3	**0.824**	0.194	0.284
Item 4	**0.765**	0.302	0.323
Item 5	**0.720**	0.279	0.404
Item 6	**0.613**	0.339	0.510
**Misinformation Beliefs**			
Item 1	**0.766**	0.189	0.378
Item 2	**0.822**	0.189	0.289
Item 3	**0.795**	0.227	0.317
Item 4	**0.783**	0.221	0.339
Item 5	**0.774**	0.248	0.340
Eigenvalue	10.682	1.775	
% Variance Explained	85.75	14.25	

Note: EFA estimated using iterated principal axis factoring. Factors are unrotated.

Of course, this result does not indicate that COVID-19 CTM beliefs and vaccine hesitancy are synonymous, nor does it mean that we should ignore beliefs in COVID-19 CTM when investigating vaccine hesitancy. It does, however, suggest that these beliefs, as measured, are so highly correlated that they are difficult to disentangle. Unidimensional structure is frequently a primary criterion to meet in the process of successfully creating and validating a psychometric scale––applying the same standard here, we could justifiably create an “anti-vax” scale composed of beliefs regarding COVID-19 CTM *and* vaccines that captures a single (category of) attitude. In a sense, then, modeling COVID-19 vaccine hesitancy based on beliefs in COVID-19 CTM may not be particularly illuminating, as such an activity amounts to an empirical tautology.

That said, we would be remiss if we failed to note that the factor analysis result could also be reflective of the model in Panel A of Fig 1. It could be the case that beliefs in COVID-19 CTM and vaccine hesitancy are so highly correlated by summer of 2021 when our data was collected because social media exposure to CTM lead to beliefs in CTM, which subsequently resulted in vaccine hesitancy and refusal. This said, we have some (empirical) reason to doubt this. The correlation between beliefs in COVID conspiracy theories and vaccine refusal is -0.38 (p<0.001). Moreover, the correlation between social media use and vaccine refusal is non-significant at 0.017 (p = 0.439). It seems to us that both the direct and indirect (through beliefs in CTM) role of media exposure are far from determinative of vaccination status, where they are correlated at all. Still, causality is difficult to assess under the best of conditions and observational data have clear limitations. This is precisely one of our central arguments: our data, as with other studies, are only capable of establishing plausibility. Unfortunately, plausibility is oftentimes not enough to adjudicate between competing theories, as in this case.

In order to further explore the plausibility of the alternative model we pose, we next turn toward comparing regression models of beliefs in CTM, vaccine hesitancy, and vaccine refusal in order to decipher which predictors are shared and which differ across constructs.

### Model comparison

Next, we examine different strategies for modeling vaccine hesitancy and behaviors. We first demonstrate how beliefs in CTM are typically modeled in the social scientific literatures that empirically examine COVID-19 behavioral intentions. The first two columns of Table 3 contain the (abbreviated) results of two such models. We include all psychological, social, and political factors described above. This group of explanatory factors is more comprehensive than any studies of which we are aware.

**Table 3:** Models of beliefs in COVID-19 CTM, vaccine hesitancy, and non-vaccination status, presented as regression coefficients with significance levels.

	(1)	(2)	(3)	(4)	(5)	(6)
	Beliefs in COVID-19 Misinformation	COVID-19 Conspiracy Theory Beliefs	Vaccinated or Planning to Vaccinate	Vaccinated or Planning to Vaccinate	Vaccine Hesitancy	Vaccine Hesitancy
COVID Misinformation			-1.169***		0.349***	
			(0.097)		(0.019)	
COVID Conspiracy Beliefs			-0.195*		0.272***	
			(0.092)		(0.019)	
Social Media Use	0.017	0.021		0.144*		-0.010
	(0.017)	(0.016)		(0.067)		(0.013)
Conspiracy Thinking	0.219***	0.295***		-0.368***		0.154***
	(0.019)	(0.017)		(0.076)		(0.015)
Science Literacy	-0.047***	-0.042***		-0.024		-0.021**
	(0.010)	(0.009)		(0.038)		(0.008)
Trust in Scientists	-0.232***	-0.191***		0.639***		-0.314***
	(0.015)	(0.014)		(0.058)		(0.012)
Partisanship	0.014	0.010		-0.069		0.021**
	(0.010)	(0.009)		(0.040)		(0.008)
Ideology	0.006	0.026*		-0.096*		-0.001
	(0.012)	(0.011)		(0.045)		(0.009)
Trump Approval	0.004***	0.005***		-0.007**		0.003***
	(0.001)	(0.001)		(0.002)		(0.000)
Machiavellianism	0.039	0.033		0.124		0.017
	(0.022)	(0.020)		(0.085)		(0.017)
Narcissism	0.016	0.036		0.231**		-0.012
	(0.021)	(0.019)		(0.081)		(0.016)
Psychopathy	0.171***	0.151***		0.092		0.032
	(0.024)	(0.022)		(0.090)		(0.018)
Conflict	0.051***	0.050***		0.005		0.010
	(0.011)	(0.010)		(0.040)		(0.009)
Perceived Victimhood	0.083***	0.084***		-0.007		0.027
	(0.021)	(0.020)		(0.083)		(0.017)
Stress	-0.037	-0.065**		-0.012		0.015
	(0.025)	(0.023)		(0.094)		(0.019)
Constant	2.260***	1.472***	1.973***	-3.591***	1.387***	3.639***
	(0.186)	(0.172)	(0.352)	(0.722)	(0.072)	(0.144)
(Pseudo) *R*^*2*^	0.551	0.605	0.304	0.301	0.561	0.559
n	2016	2016	2046	2016	2046	2016

Note: Sociodemographic controls omitted from table; see SI for estimates. Logit coefficients in columns 3–4; OLS coefficients in 1–2 and 5–6. Standard errors in parentheses.

* *p* < 0.05,

** *p* < 0.01,

*** *p* < 0.001.

We observe significant positive relationships between our two dependent variables and conspiracy thinking, ideology, Trump approval, psychopathy, perceived victimhood, and conflictual behavior. We observe significant negative relationships with science literacy, trust in scientists, and stress. Interestingly, we do not observe a relationship between beliefs in CTM and time spent on social media, contrary to the predictions of the model in Panel A of Fig 1. Hardly an empirical irregularity, this finding comports with recent work arguing that beliefs in COVID-19 CTM are more than the product of exposure alone [57]. That said, we observe significant bivariate correlations (*p*<0.001 in every case) between each variable included in Table 3 and beliefs in CTM; see the SI.

Altogether, a litany of traits, orientations, and motivations are presumably exogenous to, and theoretically formative of, beliefs in COVID-19 CTM. Indeed, most of these relationships were discovered prior to the COVID-19 pandemic and will presumably prove relevant after the COVID-19 pandemic has passed. This leads us back to our argument about modeling vaccination hesitancy and refusal: the foundational factors behind beliefs in CTM should be the focus of researchers seeking to understand vaccine hesitancy and refusal, not beliefs in specific CTM that are tethered to a particular event and are, themselves, the theoretical product of many of the same foundational factors.

Columns 3 and 5 of Table 3 present typical models of vaccine hesitancy and refusal that seek to understand the role of beliefs in COVID-19 CTM. Beliefs in CTM are treated as presumably exogenous independent variables, along with controls for sociodemographic factors (included in the models but omitted from Table 3). In both cases, as with previous work, we observe statistically significant estimates for beliefs in CTM.

While these models show evidence for hypothesized relationships, we have provided theoretical and empirical arguments about why they may not be particularly illuminative of causal relationships for researchers seeking to understand the psychological antecedents of vaccine hesitancy or working to develop strategies to encourage vaccination. We present alternative models––derived from the literature outlined above and more congruent with the model in Panel B of Fig 1—of vaccine-relevant attitudes and behaviors in columns 4 and 6. Here we have replaced beliefs in COVID-19 CTM with the predictors of those beliefs. In doing so, we observe statistically significant relationships with conspiracy thinking, science literacy, trust in scientists, ideology, and Trump approval—many of the same characteristics that foster beliefs in CTM.

More specifically, we find that conspiracy thinking, trust in scientists, and Trump approval are significant across all four models. Science literacy is a significant predictor of beliefs in CTM and vaccination status, but not vaccine hesitancy. We also find that ideology is a significant predictor of COVID-19 conspiracy beliefs and vaccine hesitancy, but not beliefs in misinformation or vaccination status. We also reiterate that social media use is unrelated to beliefs in COVID-19 CTM and vaccine hesitancy, though *positively* and statistically significantly related to vaccination status (i.e., the more social media use, the more likely one is to be vaccinated, all else equal). These results are contradictory of the model in Panel A of Fig 1 and other such models of vaccine hesitancy and refusal that situate social media use/exposure as a primary causal mechanism.

Even though beliefs in CTM and vaccine hesitancy/refusal do not share all predictors, they share many. Moreover, each substantive (i.e., non-sociodemographic) predictor in Table 3 is statistically significantly correlated with vaccine hesitancy, as well as beliefs in CTM; hence, fluctuations in statistical significance could be due to variability in the precision of measurement, multicollinearity, model specification, or true differences. We have no way of knowing, though we emphasize that this is precisely the problem with observational research that we wish to highlight: it is not capable of assessing causality, and in some case (like ours) it is not even capable of adjudicating between two plausible models. If this is the case, how can we declare a winner?

### Exploratory analysis of open-ended responses

Finally, a perusal of open-ended responses to the question, “Are there any other reasons why you have not received the COVID-19 vaccine?” offered to the unvaccinated in our survey further illustrates our argument. Some respondents—even those who point to specific CTM—mention *longstanding* views about the government, (“I am a person who is awake to the government’s agenda to depopulate the world by poisoning people with vaccines through the years. There has never been a reason to have a vaccine”), religion (“I Trust God not science and believe this is part of the Mark of the Beast”), and medicine (“Don’t believe in vaccines in general,” “I am against all vaccines they are toxic poisonous used to control population use aborted fetal cells and no vaccines protect you,” “I don’t even receive the flu shot”) that likely predate COVID-19. Such reasoning suggests that recent exposure to COVID-19 CTM is not the only cause of vaccine refusal. Furthermore, some respondents point to *factual* information as the reason for not getting vaccinated (“I see vaccinated people getting covid anyway”). Thus, beliefs in COVID-19 CTM are neither necessary for, nor strictly exogenous to, vaccine refusal.

## Discussion

The academic response to COVID-19 is unsurpassed in the speed in which it produced important findings under trying conditions. Our intention is not to diminish the great interdisciplinary strides made in explaining why individuals took part in, or eschewed, COVID-19 preventive behaviors. However, the speed of scientific discovery meant that researchers were working concurrently, rather than sequentially––this may have inadvertently slowed self-critique and challenges to the core assumptions of this rapidly expanding literature. Our investigation confronts the possibility that this literature, by not introspectively considering its modeling decisions, is (at least partially) misattributing blame for vaccine hesitancy and refusal and for other undesirable pandemic-related behaviors, as well.

Understanding the precise nature of the relationship between COVID-19 CTM and vaccine hesitancy and refusal is important not only for the sake of appropriate statistical modeling. Indeed, it speaks directly to the measures that are needed to address vaccine hesitancy. If COVID-19 CTM are merely a symptom of another problem, perhaps one that similarly impacts vaccine attitudes, then efforts to censor or correct CTM beliefs will do little to impact vaccination rates. This would also be the case if beliefs in CTM were adopted to justify preexisting preferences and behavioral intentions [65]. It may be the case, consistent with demand-driven models of media content [e.g., 66], that content producers—even those producing CTM-related content—tailor their content (i.e., supply) to match the audience’s pre-existing views (i.e., demand). Further, studies suggest that efforts aimed at removing CTM through government censorship, for example, may actually arouse suspicions, thereby *encouraging* conspiracy theory beliefs [67]. Regardless of the impact, schemes at removing or labeling content may inadvertently inhibit the free exchange of ideas that are central to democratic societies and remove from public debate ideas that are potentially true or valuable.

There was no shortage of easily accessible, high-quality information about the COVID-19 virus and vaccines during the pandemic, yet vaccine hesitancy persists. This is likely because anti-vaccine attitudes are deeply rooted, having posed a persistent threat to public health long before social media and COVID-19. We, therefore, encourage researchers to treat vaccine hesitant attitudes like other beliefs they study. Beliefs––in CTM or the dangers of vaccines––are a product of people’s motivations: the foundational ingredients of public opinion that guide which information one accepts and integrates into their belief system, and which they reject, ignore, or explain away. This fundamental argument is consistent across the literatures explaining political opinions [29], health-related beliefs [30], and beliefs in CTM [27]. In other words, simply giving people the “right” set of facts does not guarantee that they will adopt desirable beliefs or engage in advisable behaviors [68]. If we are interested in understanding the exogenous factors that promote vaccine hesitancy, we must recognize the role of people’s motivations and probe deeper than specific beliefs about a given vaccine or virus.

Many of the concerns expressed about CTM at the beginning of the pandemic are beginning to be investigated further and placed into appropriate context. As compared to some of the claims proffered by concerned researchers in early 2020, more recent work has found that online CTM is less prevalent [19,69], people engage with less online CTM [70], people share less CTM [17], CTM appears less influential [28], and echo chambers are less prominent [71,72]. Moreover, researchers are finding that online CTM and other forms of toxic rhetoric are confined to small numbers of people who exhibit unrepresentative personality traits [73,74]. While CTM, both online and offline, have been and continue to be an important social and political problem in need of attention [75–78], emerging studies suggest that it has limited scope, reach, and influence [79] and that the initial claims about an “infodemic” in 2020 may have been overstated [16,80], albeit well-intentioned.

There are, of course, limitations to our analyses. Given their cross-sectional nature, our findings only demonstrate the plausibility of our argument; this is the case for most research on COVID-19 vaccine hesitancy, a challenge that continues to obscure inferences related to the pandemic. More data, utilizing different research designs (e.g., experiments, panel data), would be invaluable for more robustly testing the plausibility of various hypotheses about causal pathways. Nevertheless, this study’s empirical evidence demonstrating the *plausibility* that COVID-19 CTM are not exogenous to vaccine hesitancy and refusal should prompt researchers to reevaluate their modeling assumptions, explore additional predictors of vaccine hesitancy, and consult the literatures on media effects, selective exposure, conspiracy theory beliefs, and vaccine attitudes for guidance on the COVID-19 case.

## Conclusion

The argument that “correlation is not causation” is neither unique nor profound, yet it is often ignored, even by the most well-intended scholars. Our findings comport with a small but growing chorus of scholars pointing out that the co-occurrence of “persistent vaccine hesitancy” and “widespread misinformation” does not indicate causation [13]. As dangerous as vaccine hesitancy is, and as bizarre as vaccine refusal may seem to members of the scientific community, socio-behavioral theories guiding human beliefs and behavior still apply. Even though beliefs in COVID-19 CTM––by virtue of their relationship with many normatively undesirable outcomes––make an attractive culprit for resistance to disease-preventive behaviors, they may not be primary, causal antecedents of those outcomes. Given increasing concern over the potential social and medical harms of CTM, we encourage alternative perspectives to studying their causes and consequences.

## Supporting information

S1 File Supporting information. This file contains supplementary information.
(PDF)
