# Supplementary material for: On the relationship between conspiracy theory beliefs, misinformation, and vaccine hesitancy
Source: PLoS One. 2022 Oct 26;17(10):e0276082. doi: 10.1371/journal.pone.0276082 (PMC9604946; doi:10.1371/journal.pone.0276082)
Supplement: S1 File — This file contains supplementary information. (PDF) [file pone.0276082.s001.pdf]

Supplemental Appendix

I. Question wording

Vaccine hesitancy.

Shapiro, Gilla K., Ovidiu Tatar, Eve Dube, Rhonda Amsel, Barbel Knauper, Anila Naz, Samara Perez, and Zeev Rosberger. "The vaccine hesitancy scale: Psychometric properties and validation." *Vaccine* 36, no. 5 (2018): 660-667.

How much do you agree with the each of the following statements about vaccinations? (each item is 1=strongly agree, 5=strongly disagree; α=0.91, *M*=2.34, *SD*=0.83)

1. Vaccines are important for my health. (Item 1)
2. Vaccines are effective. (Item 2)
3. Being vaccinated is important for the health of others in my community. (Item 3)
4. All routine vaccinations recommended by the CDC are beneficial. (Item 4)
5. New vaccines carry more risks than older vaccines. (reversed, Item 5)
6. The information I receive about vaccines from the CDC is reliable and trustworthy. (Item 6)
7. Getting vaccines is a good way to protect me from disease. (Item 7)
8. Generally, I do what my doctor or healthcare provider recommends about vaccines for me. (Item 8)
9. I am concerned about serious adverse effects of vaccines. (reversed, Item 9)
10. I do not need vaccines for diseases that are not common anymore. (reversed, Item 10)

Science literacy.

Okamoto, S., Niwa, F., Shimizu, K., & Sugiman, T. (2001). The 2001 Survey for Public Attitudes Towards and Understanding of Science and Technology in Japan. *NISTEP Report, 72.*

To the best of your knowledge, are the following statements true or false? (1=correct; 0=incorrect)

1. The continents on which we live have been moving their location for millions of years and will continue to move in the future. (True)
2. All radioactivity is manmade. (False)
3. The center of the Earth is very hot. (True)
4. The oxygen we breathe comes from plants. (True)
5. Lasers work by focusing sound waves. (False)
6. Electrons are smaller than atoms. (True)
7. Antibiotics kill viruses as well as bacteria. (False)
8. The earliest humans lived at the same time as the dinosaurs. (False)
9. Human beings, as we know them today, developed from earlier species of animals. (True)
10. It is the father's gene that decides whether the baby is a boy or a girl. (True)
11. Radioactive milk can be made safe by boiling it. (False)

Trust in science.

Merkley, E. (2020). Anti-Intellectualism, Populism, and Motivated Resistance to Expert Consensus. Public Opinion Quarterly. doi:10.1093/poq/nfz053

Please tell us how much you trust each of the groups of people below (each item is 1=distrust a lot, 7=trust a lot; α=0.84, *M*=5.24, *SD*=1.23):

1. Scientists
2. Doctors
3. Public health officials

Conspiracy thinking.

Enders, Adam M., Joseph E. Uscinski, Michelle I. Seelig, Casey A. Klofstad, Stefan Wuchty, John R. Funchion, Manohar N. Murthi, Kamal Premaratne, and Justin Stoler. "The relationship between social media use and beliefs in conspiracy theories and misinformation." *Political behavior* (2021): 1-24.

Please tell us how much you agree or disagree with each of the statements below (each item is 1=strongly disagree, 5=strongly agree; α=0.86, *M*=3.06, *SD*=1.02):

1. Much of our lives are being controlled by plots hatched in secret places.
2. Even though we live in a democracy, a few people will always run things anyway.
3. The people who really 'run' the country, are not known to the voters.
4. Big events like wars, the current recession, and the outcomes of elections are controlled by small groups of people who are working in secret against the rest of us.

Machiavellianism.

Jonason, P. K., & Webster, G. D. (2010). The dirty dozen: A concise measure of the dark triad. Psychological assessment, 22(2), 420-432.

Please tell us how much you agree or disagree with each of the statements below (each item is 1=strongly disagree, 5=strongly agree; α=0.87, *M*=2.17, *SD*=0.99):

1. I tend to manipulate others to get my way.
2. I have used deceit or lied to get my way.
3. I have used flattery to get my way.
4. I tend to exploit others towards my own end.

Narcissism.

Jonason, P. K., & Webster, G. D. (2010). The dirty dozen: A concise measure of the dark triad. Psychological assessment, 22(2), 420-432

Please tell us how much you agree or disagree with each of the statements below (each item is 1=strongly disagree, 5=strongly agree; α=0.87, *M*=2.51, *SD*=1.00):

1. I tend to want others to admire me.
2. I tend to want others to pay attention to me.
3. I tend to seek prestige or status.
4. I tend to expect special favors from others.

Psychopathy.

Jonason, P. K., & Webster, G. D. (2010). The dirty dozen: A concise measure of the dark triad. Psychological assessment, 22(2), 420-432.

Please tell us how much you agree or disagree with each of the statements below (each item is 1=strongly disagree, 5=strongly agree; α=0.83, *M*=2.15, *SD*=0.89):

1. I tend to lack remorse.
2. I tend to be unconcerned with the morality of my actions.
3. I tend to be callous or insensitive.
4. I tend to be cynical.

Conflict scale.

Conrad, Kendon J., Barth B. Riley, Karen M. Conrad, Ya-Fen Chan, and Michael L. Dennis. "Validation of the Crime and Violence Scale (CVS) against the Rasch measurement model including differences by gender, race, and age." Evaluation review 34, no. 2 (2010): 83-115.

During the past 12 months, have you done the following things when having a disagreement with another person? (cumulative scale ordered as follows):

1. Insulted, swore, or cursed at someone
2. Threatened to hit or throw something at another person
3. Pushed, grabbed, or shoved someone
4. Actually threw something at someone
5. Slapped another person
6. Hit or tried to hit anyone with an object
7. Kicked, bit, or hit someone
8. Beat up someone
9. Threatened anyone with knife or gun
10. Actually used a knife or gun on someone

Victimhood.

Armaly, M. T., & Enders, A. M. (2021). 'Why Me?' The Role of Perceived Victimhood in American Politics. Political Behavior. doi:10.1007/s11109-020-09662-x

Please tell us how much you agree or disagree with each of the statements below (each item is 1=strongly disagree, 5=strongly agree; α=0.87, *M*=2.78, *SD*=0.97):

1. I rarely get what I deserve in life.
2. Great things never come to me.
3. I usually have to settle for less.
4. I never seem to get an extra break.

Stress.

Cohen, Sheldon, Tom Kamarck, and Robin Mermelstein. "A global measure of perceived stress." Journal of health and social behavior (1983): 385-396.

In the past month, how often have you (each item is 1=never, 5=very often; α=0.83, *M*=2.15, *SD*=0.89):

1. Felt that you were unable to control the important things in your life?
2. Felt confident about your ability to handle your personal problems?
3. Felt that things were going your way?
4. Felt difficulties were piling up so high that you could not overcome them?

Social media use.

Enders, Adam M., Joseph E. Uscinski, Michelle I. Seelig, Casey A. Klofstad, Stefan Wuchty, John R. Funchion, Manohar N. Murthi, Kamal Premaratne, and Justin Stoler. "The relationship between social media use and beliefs in conspiracy theories and misinformation." *Political behavior* (2021): 1-24.

Thinking about the social media you use, how often in a typical week do you visit or use: 1) Facebook, 2) Twitter, 3) Instagram, 4) YouTube (each item is 1=not at all, 5=everyday; α=0.70, *M*=2.09, *SD*=1.17):

1. Not at all
2. Once a month or less
3. Several times a month
4. Several times a week
5. Everyday

Trump Thermometer.

Uscinski, Joseph E., Adam M. Enders, Michelle I. Seelig, Casey A. Klofstad, John R. Funchion, Caleb Everett, Stefan Wuchty, Kamal Premaratne, and Manohar N. Murthi. "American politics in two dimensions: Partisan and ideological identities versus anti-establishment orientations." American Journal of Political Science 65, no. 4 (2021): 877-895.

Next, we would like to know your feelings about public figures. Please rate the names below using the "feeling thermometer" slider bars. Ratings between 50 and 100 degrees mean that you feel favorable toward the name. Ratings between 0 and 50 degrees mean that you do not feel favorable toward the name.

Ideology. Where would you place yourself on a scale that goes from "very liberal" to "very conservative"?

1. Extremely liberal
2. Liberal
3. Slightly liberal
4. Moderate
5. Slightly conservative
6. Conservative
7. Extremely conservative

Partisanship. (standard, two-question branching measurement strategy)

1. Strong Democrat
2. Weak Democrat
3. Lean Democrat
4. True Independent
5. Lean Republican
6. Weak Republican
7. Strong Republican

Religiosity. Aside from weddings and funerals, how often do you attend religious services?

1. Never
2. Seldom
3. A few times a year
4. Once or twice a month
5. Once a week
6. More than once a week

Born again. Would you describe yourself as a "born-again" or evangelical Christian, or not?

0. No
1. Yes

Denomination. What is your present religion, if any?

1. Protestant
2. Roman Catholic
3. Mormon (coded as "Other Christian")
4. Eastern or Greek Orthodox (coded as "Other Christian")
5. Jewish (coded as "Other Religion")
6. Muslim (coded as "Other Religion")
7. Buddhist (coded as "Other Religion")
8. Hindu (coded as "Other Religion")
9. Atheist (coded as "None")
10. Agnostic (coded as "None")
11. Nothing in particular (coded as "None")
12. Something else

Sociodemographics.

1. Educational attainment (6-point scale, 1=No high school degree, 5=post-grad degree)
2. Age (age in years, 18–95)
3. Household income (7-point scale, 1=$24,999 or less, 7=200,000 or more)
4. Gender (0=male, 1=female)
5. Race (Black: 0=not Black, 1=Black; Hispanic: 0=not Hispanic, 1=Hispanic)

Socioeconomic status.

Adler, Nancy, and Judith Stewart. "The MacArthur scale of subjective social status." San Francisco: MacArthur Research Network on SES & Health (2007).

Think of this ladder as representing where people stand in their communities. People define community in different ways; please define it in whatever way is most meaningful to you. At the top of the ladder are the people who have the highest standing in their community. At the bottom are the people who have the lowest standing in their community.
Where would you place yourself on this ladder? Please click on the rung where you think you stand at this time in your life, relative to other people in your community. (coded 1=lowest to 10=highest)

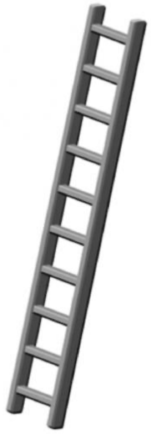

II. Sample composition

Table A1: Sociodemographic information about July 2021 sample, compared to 2010 U.S. Census estimates.

Characteristic	July 2021	2010 Census Estimate
Age (median)	46	38
High school degree (%)	97	88
Some college or more (%)	73	59
Female (%)	52	51
Household income (median)	$50,000–$74,999	$49,445
Race:		
White (%)	72	72
Black (%)	16	13
Hispanic (%)	18	16
n	2,065	

III. Scree plot

Figure A1: Scree plot of eigenvalues from exploratory factor analysis against number of factors.

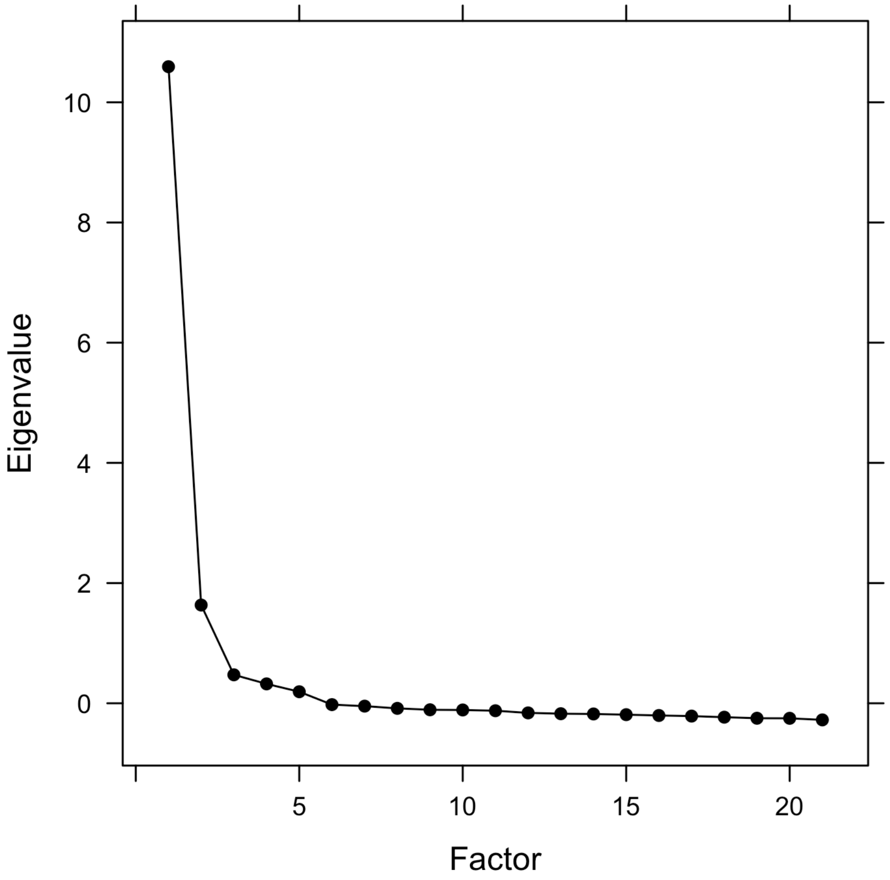

IV. Correlations between predictors beliefs in CTM and vaccine hesitancy

Table A2: Pearson product-moment correlations.

	Beliefs in COVID-19 Misinformation	COVID-19 Conspiracy Theory Beliefs	Vaccine Hesitancy
Conspiracy Thinking	0.531^{***}	0.596^{***}	0.495^{***}
Science Literacy	-0.326^{***}	-0.330^{***}	-0.240^{***}
Trust in Scientists	-0.455^{***}	-0.452^{***}	-0.635^{***}
Partisanship	0.221^{***}	0.274^{***}	0.296^{***}
Ideology	0.157^{***}	0.224^{***}	0.221^{***}
Trump Approval	0.396^{***}	0.481^{***}	0.411^{***}
Social Media for News	0.207^{***}	0.187^{***}	0.076^{***}
Machiavellianism	0.313^{***}	0.312^{***}	0.160^{***}
Narcissism	0.246^{***}	0.265^{***}	0.069^{**}
Psychopathy	0.376^{***}	0.375^{***}	0.208^{***}
Perceived Victimhood	0.295^{***}	0.281^{***}	0.217^{***}
Stress	0.239^{***}	0.190^{**}	0.230^{***}
Conflict	0.343^{***}	0.339^{***}	0.195^{***}

Note: * $p < 0.05$, ** $p < 0.01$, *** $p < 0.001$

V. Full regression results, Table 3

Table A3: OLS regressions of beliefs in COVID-19 conspiracy theories and misinformation on psychological, social, and political factors.

	Beliefs in COVID-19 Misinformation	COVID-19 Conspiracy Theory Beliefs
Female	0.121***	0.062
	(0.035)	(0.032)
Age	-0.011***	-0.006***
	(0.001)	(0.001)
Education	-0.026*	-0.034**
	(0.013)	(0.012)
Perceived Social Status	0.011	0.018*
	(0.008)	(0.008)
Religiosity	0.118***	0.131***
	(0.016)	(0.015)
Evangelical	-0.026	-0.097*
	(0.051)	(0.047)
Latinx	-0.116**	-0.013
	(0.045)	(0.042)
Black	0.106*	0.095*
	(0.049)	(0.046)
Asian	-0.035	0.045
	(0.065)	(0.060)
Native American	0.052	-0.058
	(0.097)	(0.089)
Other Race	0.091	0.015
	(0.084)	(0.078)
Conspiracy Thinking	0.219***	0.295***
	(0.019)	(0.017)
Science Literacy	-0.047***	-0.042***
	(0.010)	(0.009)
Trust in Scientists	-0.232***	-0.191***
	(0.015)	(0.014)
Partisanship	0.014	0.010
	(0.010)	(0.009)
Ideology	0.006	0.026*
	(0.012)	(0.011)
Trump Approval	0.004***	0.005***
	(0.001)	(0.001)
Social Media for News	0.017	0.021
	(0.017)	(0.016)
Machiavellianism	0.039	0.033
	(0.022)	(0.020)

Narcissism	0.016	0.036
	(0.021)	(0.019)
Psychopathy	0.171***	0.151***
	(0.024)	(0.022)
Perceived Victimhood	0.083***	0.084***
	(0.021)	(0.020)
Stress	-0.037	-0.065**
	(0.025)	(0.023)
Conflict	0.051***	0.050***
	(0.011)	(0.010)
Constant	2.260***	1.472***
	(0.186)	(0.172)
R^2	0.551	0.605
n	2016	2016

Note: OLS coefficients with standard errors in parentheses. White is the reference race category.
* $p < 0.05$, ** $p < 0.01$, *** $p < 0.001$

VI. Full regression results, Table 4

Table A4: Models of vaccine hesitancy and non-vaccination status.

	(1) Vaccinated or Planning to Vaccinate	(2) Vaccinated or Planning to Vaccinate	(3) Vaccine Hesitancy	(4) Vaccine Hesitancy
Female	-0.742***	-0.793***	0.037	0.074**
	(0.130)	(0.136)	(0.026)	(0.027)
Age	0.004	0.042***	0.000	-0.007***
	(0.004)	(0.005)	(0.001)	(0.001)
Education	0.278***	0.211***	-0.040***	-0.032**
	(0.049)	(0.051)	(0.009)	(0.010)
Perceived Social Status	0.118***	0.041	-0.028***	-0.001
	(0.029)	(0.031)	(0.006)	(0.007)
Religiosity	0.268***	-0.043	-0.050***	0.027*
	(0.060)	(0.062)	(0.012)	(0.013)
Evangelical	-0.289	0.129	0.072	-0.035
	(0.187)	(0.189)	(0.038)	(0.039)
Latinx	0.822***	0.525**	0.008	0.030
	(0.180)	(0.181)	(0.034)	(0.035)
Black	-0.020	-0.516**	-0.009	0.101**
	(0.169)	(0.182)	(0.035)	(0.038)
Asian	1.468***	1.146***	-0.052	-0.010
	(0.316)	(0.325)	(0.049)	(0.051)
Native American	-0.427	-0.660	0.138	0.131
	(0.345)	(0.340)	(0.073)	(0.075)
Other Race	-0.265	-0.169	0.071	0.051
	(0.307)	(0.323)	(0.063)	(0.065)
COVID Misinformation	-1.169***		0.349***	
	(0.097)		(0.019)	
COVID Conspiracy Beliefs	-0.195*		0.272***	
	(0.092)		(0.019)	
Social Media for News		0.144*		-0.010
		(0.067)		(0.013)
Conspiracy Thinking		-0.368***		0.154***
		(0.076)		(0.015)
Science Literacy		-0.024		-0.021**
		(0.038)		(0.008)
Trust in Scientists		0.639***		-0.314***
		(0.058)		(0.012)
Partisanship		-0.069		0.021**
		(0.040)		(0.008)
Ideology		-0.096*		-0.001
		(0.045)		(0.009)

Trump Approval		-0.007^{**}		0.003^{***}
		(0.002)		(0.000)
Machiavellianism		0.124		0.017
		(0.085)		(0.017)
Narcissism		0.231^{**}		-0.012
		(0.081)		(0.016)
Psychopathy		0.092		0.032
		(0.090)		(0.018)
Perceived Victimhood		-0.007		0.027
		(0.083)		(0.017)
Stress		-0.012		0.015
		(0.094)		(0.019)
Conflict		0.005		0.010
		(0.040)		(0.009)
Constant	1.973^{***}	-3.591^{***}	1.387^{***}	3.639^{***}
	(0.352)	(0.722)	(0.072)	(0.144)
(Pseudo) R^2	0.304	0.301	0.561	0.559
n	2046	2016	2046	2016

Note: Logit coefficients in 1-2; OLS coefficients in 3-4. Standard errors in parentheses. White is the reference race category. * $p < 0.05$, ** $p < 0.01$, *** $p < 0.001$

VII. Model results with alternative measure of vaccination status

Table A5: Models of non-vaccination status where only those who have not been vaccinated and are completely unwilling to be vaccinated are coded as 0.

	(1) Vaccinated	(2) Vaccinated
Female	-0.668***	-0.701***
	(0.123)	(0.127)
Age	0.011**	0.039***
	(0.004)	(0.005)
Education	0.243***	0.190***
	(0.046)	(0.047)
Perceived Social Status	0.111***	0.049
	(0.027)	(0.030)
Religiosity	0.200***	-0.041
	(0.057)	(0.058)
Evangelical	-0.204	0.119
	(0.178)	(0.179)
Latinx	0.781***	0.535**
	(0.167)	(0.167)
Black	-0.047	-0.422*
	(0.162)	(0.171)
Asian	1.343***	1.102***
	(0.283)	(0.288)
Native American	-0.371	-0.495
	(0.330)	(0.329)
Other Race	-0.209	-0.168
	(0.293)	(0.305)
COVID Misinformation	-1.032***	
	(0.091)	
COVID Conspiracy Beliefs	-0.178*	
	(0.088)	
Social Media for News		0.015
		(0.063)
Conspiracy Thinking		-0.321***
		(0.071)
Science Literacy		0.011
		(0.035)
Trust in Scientists		0.568***
		(0.055)
Partisanship		-0.041
		(0.037)
Ideology		-0.101*
		(0.042)

Trump Approval		-0.006**
		(0.002)
Machiavellianism		0.074
		(0.079)
Narcissism		0.196*
		(0.076)
Psychopathy		0.063
		(0.084)
Perceived Victimhood		-0.005
		(0.078)
Stress		-0.016
		(0.089)
Conflict		0.016
		(0.038)
Constant	1.300***	-3.444***
	(0.329)	(0.677)
(Pseudo) R^2	0.276	0.267
n	2046	2016

Note: Logit coefficients. Standard errors in parentheses.
* $p < 0.05$, ** $p < 0.01$, *** $p < 0.001$
